# Cochlear implant in type 2 neurofibromatosis: an option for better hearing rehabilitation

**DOI:** 10.1590/S1808-86942011000400022

**Published:** 2015-10-19

**Authors:** Oswaldo Laércio M. Cruz, Eduardo A.S. Vellutini

**Affiliations:** 1 Associate professor, affiliated professor - Otorhinolaryngology Department - UNIFESP; 2 Doctoral degree in neurosurgery - FMUSP. Neurosurgeon - DFV

**Keywords:** cochlear implantation, hearing loss, sensorineural, neurofibromatosis 2

## INTRODUCTION

Type 2 neurofibromatosis (NF2) is an autosomal dominant disease with a frequency of 0.5/100,000 inhabitants; it manifests mainly as bilateral vestibular schwannomas and bilateral profound hearing loss.

Brainstem implants have been the main hearing rehabilitation method in these patients.^1^ Although this approach is a significant advance in the treatment of these patients, the qualitative results of brainstem implants are more limited than those of cochlear implants.^2^ Loss of electrical stimulus processing in the cochlea (by cells in the spiral ganglion and cochlear nerve) and the difficulty of positioning electrodes in the fourth ventricle explain a poorer hearing performance with brainstem implants.^3^

The past decades have witnessed refinements in the results of surgeries for the treatment of vestibular schwannomas. The possibility of preserving the cochlear nerve in neurofibromatosis cases makes it feasible to fit cochlear implants for improved auditory rehabilitation.^2,^^4,^^5^

We present the results attained in a patients with neurofibromatosis in whom the neoplasm was removed and a cochlear implant was placed simultaneously. Based on a review of the literature, this is the first documented case of this procedure in Brazil.

## CASE REPORT

**Figure 1:**
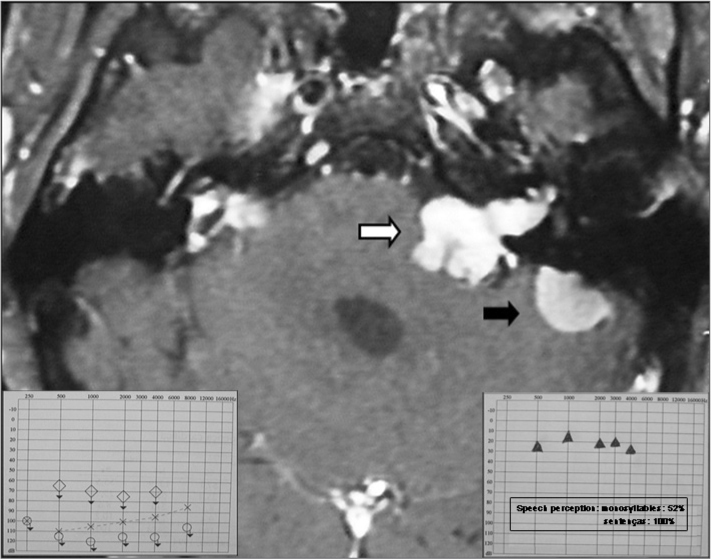
Magnetic resonance imaging (T1 contrast) showing a vestibular schwannoma to the left (white arrow) associated with a meningioma on the medial aspect of the left petrous bone (black arrow). Note also small manipulation remains in the right pontocerebellar angle. On the lower left detail, see preoperative audiometry. On the lower right detail see audiometry 6 months after the cochlear implant.

HC, a white male student aged 23 years, diagnosed with neurofibromatosis type 2, developed significant hearing loss to the right, which was associated with tumor growth in that side. The tumor was removed surgically in 2005, but the patient progressed to total hearing loss. In 2008, the patients noticed marked hearing loss to the left, diagnosed as profound hearing loss due to growth of the ipsilateral tumor (Fig. 1, lower left detail). Imaging showed also a meningioma located posterior to the inner auditory canal (Fig. 1).

Simultaneous surgery and brainstem implant were indicated. The patient did not want the brainstem implant, and it was agreed that if the cochlear nerve remained intact, a cochlear implant would be fitted.

Translabyrinthine surgery was uneventful; the meningioma and the vestibular schwannoma were removed, and the facial and cochlear nerves were preserved; a cochlear implant was placed. Intraoperative neural telemetry confirmed consistent responses in all electrodes.

The facial nerve remained functional.

Imaging for control purposes demonstrated that the tumors had been adequately removed. After 6 months of cochlear implant use, the pure tone threshold was around 30 dB, monosyllable discrimination was 56%, and open set sentence discrimination was 100% (Fig. 1, lower right detail).

## DISCUSSION

Treating patients with neurofibromatosis type 2 remains a difficult task. Fortunately, satisfactory results have currently been attained in the treatment of this disease, and patients have become more concerned with their quality of life. Approaches to restore hearing should always be taken into account when treating neurofibromatosis type 2, as bilateral profound hearing loss is an almost universal event in this condition. Brainstem implants have been the main choice for many years, and have yielded interesting but limited results in language discrimination.^1,^^2,^^4,^^5^ Reports of cochlear implant use in patients with neurofibromatosis type 2 have shown that they are superior to brainstem implants for increasing auditory abilities and for sustaining these results in the long term.^2,^^5^ Therefore, cochlear implants have become an option in such circumstances. The indispensable condition for this approach is that the anatomy and function of the cochlear nerve be preserved. From a surgical perspective, anatomical preservation may be attained with careful microsurgical techniques, and preferably operating smaller tumors. This should be taken into account in the treatment of neurofibromatosis; thus, surgery for less developed tumors with milder forms of hearing loss may be considered if the cochlear nerve is to be preserved - especially if the possibility of contralateral auditory rehabilitation is absent. Determining the functional viability of the cochlear nerve is more complex. Electric stimulation tests of the promontory are still employed, but are rather subjective.^2^ The possibility of carrying out electrically evoked auditory potentials seems to us more adequate, but this test is not routinely available.^6^

Finally, cochlear implants are safer than brainstem implants in terms of the eventual adverse effects of electrical stimulation; they are also less costly, and it is more practical to maintain its programming and electronic components. Furthermore, cochlear implants may be used in patients that live far from tertiary care or high complexity healthcare centers.

Our case of a young patients with bilateral hearing loss that had already been operated, without hearing rehabilitation, benefited significantly from a cochlear implant fitter immediately after removing his second vestibular schwannoma, enjoying improved quality of life.

## FINAL COMMENTS

Fitting a cochlear implant is an approach to be considered in the surgical planning of neurofibromatosis type 2 patients with bilateral schwannomas. This may be justified because of the superior auditory results that this technique provides compared to brainstem implants, as well as more safety and easier maintenance and programming.
